# Unraveling a molecular determinant for clathrin-independent internalization of the M2 muscarinic acetylcholine receptor

**DOI:** 10.1038/srep11408

**Published:** 2015-06-22

**Authors:** Min Wan, Wenhua Zhang, Yangli Tian, Chanjuan Xu, Tao Xu, Jianfeng Liu, Rongying Zhang

**Affiliations:** 1 Key Laboratory of Molecular Biophysics of the Ministry of Education, College of Life Science and Technology, Huazhong University of Science and Technology, Wuhan, Hubei, China; 2 National Laboratory of Biomacromolecules, Institute of Biophysics, Chinese Academy of Sciences, Beijing, China

## Abstract

Endocytosis and postendocytic sorting of G-protein-coupled receptors (GPCRs) is important for the regulation of both their cell surface density and signaling profile. Unlike the mechanisms of clathrin-dependent endocytosis (CDE), the mechanisms underlying the control of GPCR signaling by clathrin-independent endocytosis (CIE) remain largely unknown. Among the muscarinic acetylcholine receptors (mAChRs), the M4 mAChR undergoes CDE and recycling, whereas the M2 mAChR is internalized through CIE and targeted to lysosomes. Here we investigated the endocytosis and postendocytic trafficking of M2 mAChR based on a comparative analysis of the third cytoplasmic domain in M2 and M4 mAChRs. For the first time, we identified that the sequence ^374^KKKPPPS^380^ servers as a sorting signal for the clathrin-independent internalization of M2 mAChR. Switching ^374^KKKPPPS^380^ to the i3 loop of the M4 mAChR shifted the receptor into lysosomes through the CIE pathway; and therefore away from CDE and recycling. We also found another previously unidentified sequence that guides CDE of the M2 mAChR, ^361^VARKIVKMTKQPA^373^, which is normally masked in the presence of the downstream sequence ^374^KKKPPPS^380^. Taken together, our data indicate that endocytosis and postendocytic sorting of GPCRs that undergo CIE could be sequence-dependent.

The endocytic pathway strongly controls the activity of G-protein-coupled receptors (GPCRs)^1^,^2^,^3^. Many GPCRs are subject to the classical clathrin-endocytic pathway, which involves dynamin, β-arrestin, and clathrin-coated pits^2^,^4^. In contrast, some GPCRs, such as endothelin receptor type A^5^,^6^, angiotensin II type 1A receptor^7^,^8^, and M2 muscarinic receptor^9^,^10^ are internalized via clathrin-independent mechanisms. After internalization, molecular sorting of GPCRs through divergent membrane pathways can result in highly different functional consequences^11^. Elucidating the mechanisms that determine the specificity of GPCR membrane traffic in the endocytic pathway will offer new insights into the regulation of GPCR functions^3^.

Muscarinic acetylcholine receptors (mAChRs) are an important subfamily of class A GPCRs. Of the five mAChR subtypes, the M2 and M4 mAChRs couple predominantly to Gi/Go-type G proteins^12^. Distinct from most GPCRs, mAChRs possess a long third intracellular loop (the i3 loop, positioned between transmembrane domains TM5 and TM6) with more than 180 amino acid residues^13^. This loop appears to be extensively involved in the regulation of the receptors, including involvement in agonist-promoted internalization^14^,^15^,^16^,^17^, downregulation^15^, and phosphorylation^18^. Although the M2 and M4 mAChRs are analogous and share several signal transduction pathways, such as mediating inhibiting adenylyl cyclase, increasing [Ca^2+^]_i_ level, activating phospholipase C, and regulating mitogen-activated protein kinase (MAPK) signal^19^, their endocytosis and postendocytic trafficking routes are distinct. After agonist induction, the M2 mAChR is internalized via a clathrin-independent endocytosis (CIE) pathway, suggested to be regulated by Arf6^9^ and then targeted to lysosomes for degradation^20^. In contrast, the M4 mAChR is internalized through the clathrin-dependent endocytosis (CDE) pathway^3^ and then recycled back to the plasma membrane, a process that involves Myosin Vb and Rab11^21^. The M2 and M4 mAChRs show 58% sequence homology; however, alignment of the amino acid sequences within the i3 loop regions of each receptor shows that their composition is divergent, with a shared identity of only 24%. Therefore, an open question is whether the distinction in endocytosis and postendocytic trafficking observed between the M2 and M4 mAChRs is due to differences in their i3 loops, if so, identification of the determinant sequence and the functional consequences on the mAChRs’ signal transduction would be important.

In the M4 mAChR, Hashimoto *et al*. has previously identified a segment of 21 amino acid residues in the i3 loop that is involved in agonist-dependent internalization and recycling of the M4 receptor, and presumably is recognized by other proteins^22^. In the present study, we investigated the sorting sequences within the i3 loops of M2 and M4 mAChRs with a specific focus on their distinct endocytic and postendocytic trafficking properties. Fluorescent microscopy combined with ELISA was used to examine the endocytosis and postendocytic trafficking of a set of M2- and M4-based deletion mutants or hybrid chimeras. Our results showed that analogous domains in the membrane-proximal carboxyl terminus of the i3 loops specify the relationship between the CIE pathway and M2 mAChR *versus* that between the CDE pathway and M4 mAChR, but these sequences are differentially required for the postendocytic trafficking of the mAChR subtypes. Taken together, our results identify a novel sorting sequence that determines CIE of the M2 mAChR and probably also contributes to the subsequent trafficking into lysosomes. This finding improves our understanding of the divergent mechanisms involved in the regulation of GPCR trafficking.

## Results

### The extent and rate of internalization of M2 and M4 mAChRs

**Figure 1:**
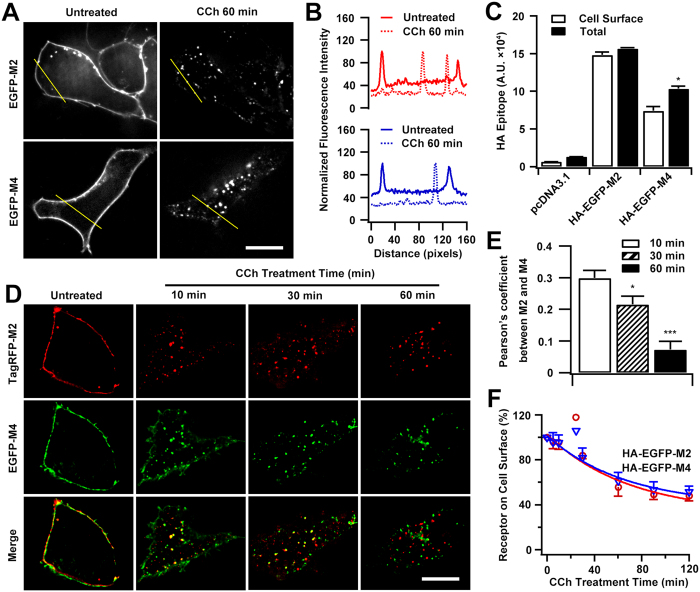
Time courses of CCh-stimulated internalization of M2 and M4 mAChRs. (**A**) Representative live imaging of HEK293 cells expressing EGFP-M2 or EGFP-M4 before or after CCh treatment. Scale bar, 10 μm. (**B**) Linear profile analysis showed the plasma membrane-associated M2 or M4 signals were translocated inside of cells after CCh (100 μM) treatment for 60 min, as depicted in *A*. (**C**) Surface and total expression of mAChRs. HEK293 cells were transfected with HA-EGFP-M2 or HA-EGFP-M4 and measured by ELISA using a monoclonal anti-HA antibody, pcDNA3.1 was used as control. (**D**) Confocal micrographs of cells co-expressing TagRFP-M2 and EGFP-M4, in the absence or presence of CCh for the indicated time courses. Images representative of different conditions are shown from at least 15 cells visualized per condition and over three separate experiments. Scale bar, 10 μm. (**E**) The levels of colocalization between TagRFP-M2 and EGFP-M4 depicted in *D* were determined by Pearson’s coefficient as described in ′′*Methods*′′. (**F**) Time-dependency of CCh-induced internalization of M2 and M4 receptors. Internalization for cells expressing HA-EGFP-M2 or HA-EGFP-M4 was measured by ELISA. Shown is the percentage of residual surface receptors following agonist exposure for 5, 10, 30, 60, 90 or 120 min with respect to that at time point of 0. Mean ± SEM of at least three independent experiments is shown for each time points. The kinetics curves were generated by single exponential fitting. *p < 0.05, ***p < 0.001.

To visualize internalization, fluorophore-tagged M2 and M4 mAChRs were developed. An N-terminal cleavable signal peptide preceding the EGFP or TagRFP sequence, which was previously demonstrated to be efficient for the functional expression of various mAChR subtypes^23^,^24^, was used. The internalization of the M2 and M4 mAChRs in HEK293 cells was examined using imaging and ELISA techniques. Both mAChRs were located at the cell surface before carbachol (CCh) stimulation (Fig. 1A,B). ELISA results confirmed that most of the exogenously expressed M2 and M4 mAChRs resided at the cell surface, with relative surface expression (surface/total protein) of 95% and 72% for M2 and M4, respectively (Fig. 1C). This distribution pattern presumably arises from the inert constitutive endocytosis of the mAChRs^25^. Upon CCh stimulation, however, the plasma membrane-associated mAChR molecules were substantially diminished and the fluorescent puncta appeared inside the cells (Fig. 1A,B).

EGFP-M4 and TagRFP-M2 mAChRs were cotransfected to examine their internalization kinetics (Fig. 1D). After a 10-min exposure to CCh, both mAChRs moved from the cell surface and accumulated in vesicular, endosome-like structures in the cytosol and displayed obvious colocalization with a Pearson’s coefficient of 0.30 (Fig. 1E). However, prolonged CCh exposure (30 min) led to segregation of the intracellular M2 and M4 mAChRs, as indexed by the reduced overlap of fluorescent signals. When CCh stimulation was prolonged to 60 min, the M2 and M4 mAChRs further segregated from each other, with only a small amount of colocalization observed (Pearson’s coefficient of 0.07). The surface receptor levels examined by ELISA also demonstrated the time-dependent loss of cell surface-associated mAChRs (Fig. 1F). The half-time (t_1/2_) for internalization of M2 and M4 mAChR was 48 and 46 min, respectively; and a ~50% loss of surface expression for both mAChRs was observed at 2 h. These results are consistent with previous report^26^.

Taken together, these results demonstrate that targeting to the plasma membrane using the signal sequence had no effect on the endocytic ability and postendocytic transport of the fluorophore-tagged mAChRs; therefore, the constructs used could be applied in further investigations of mAChRs trafficking. In addition, the decay curve from the assay of surface receptor expression suggested that M2 and M4 mAChRs are internalized to the same extent and at similar rates during 2 h agonist stimulations.

### Internalized M2 mAChRs were first transported into classical early endosomes

**Figure 2:**
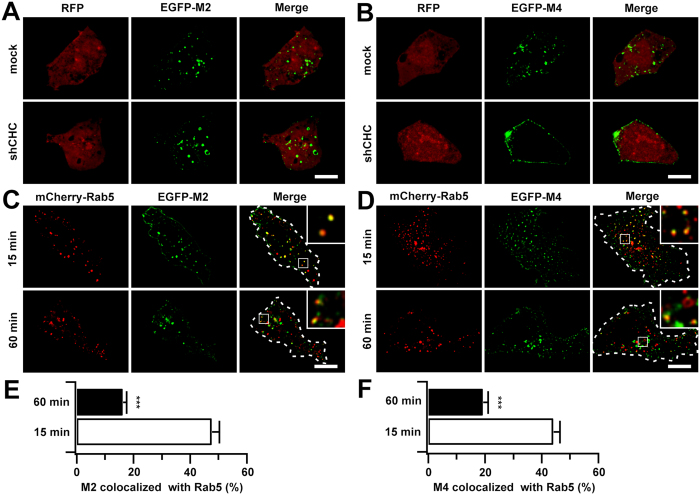
M2 and M4 mAChRs were transported to Rab5-positive early endosomes via different endocytic pathways. (**A,B**) HEK293 cells expressing EGFP-M2 (**A**) or EGFP-M4 (**B**) receptors were transfected with mock or CHC shRNA and were assessed for CCh-stimulated internalization by confocal microscopy. Shown only are cells treated for 60 min of CCh (100 μM). (**C,D**) HEK293 cells were co-transfected with EGFP-M2 (**C**) or EGFP-M4 (**D**) and mCherry-Rab5. Representative confocal images show the localization of internalized M2 or M4 receptors with Rab5 following 15 or 60 min stimulation of CCh (100 μM). Shown only are cells treated for various time of CCh. Cell contours were outlined and insets showed the magnified boxed regions. Scale bars, 10 μm. (**E,F**) The levels of colocalization of EGFP-M2 (**E**) and EGFP-M4 (**F**) with Rab5 depicted in C and *D* were determined by Manders coefficient as described in ′′*Methods*′′. ***p < 0.001.

Prior evidence suggests that M2 and M4 mAChRs undergo CIE and CDE, respectively, in HEK293 cells^27^. Here, we investigated the role of the clathrin pathway in the internalization of M2 and M4 mAChRs by using shRNA methods to knockdown clathrin heavy chain. Uptake of the Alexa 568-tagged transferrin (Tfn) was examined to evaluate silencing efficiency (Fig. S1)^28^. CCh-stimulated internalization of M2 or M4 mAChR was then assessed using the CHC-depleted cells (Fig. 2A,B). The internalization efficiency of the M2 mAChR was not affected in CHC-depleted cells, supporting the hypothesis that the CIE mechanism was preferred by M2 mAChRs (Fig. 2A); however, internalization of M4 mAChRs was almost completely blocked due to their association with CDE (Fig. 2B).

One of the central requirements for understanding the role of trafficking in GPCR regulation is identification of the intracellular organelles through which the receptors move. Given that following internalization, the M2 and M4 mAChRs converged in the earlier stage of postendocytic trafficking and were subsequently segregated (Fig. 1D), we examined the traffic of M2 and M4 mAChRs through early endosomes at different time points after agonist induction (Fig. 2C,D). We found that not only the clathrin-dependent M4 mAChR (44%) but also the clathrin-independent M2 mAChR (48%) were transported to Rab5-positive early endosomes 15 min after internalization, which is consistent with the fusion of separate endocytic vesicles with the early endosomal compartment immediately after their internalization. At the 60 min post-internalization point, both receptors moved out of the early endosomes, with only 19% and 16% of M4 and M2 remaining, respectively (Fig. 2E,F). These results suggest that, although M2 mAChRs are internalized via CIE pathways, they are first transported to the classical Rab5-positive early endosomes where they converge with the clathrin cargoes, similar to M4 mAChRs. In these endosomes, they appear to be sorted and further transported along different routes.

### Identification of the i3 loop-located domains that determine the modes of internalization of the M4 and M2 mAChRs

**Figure 3:**
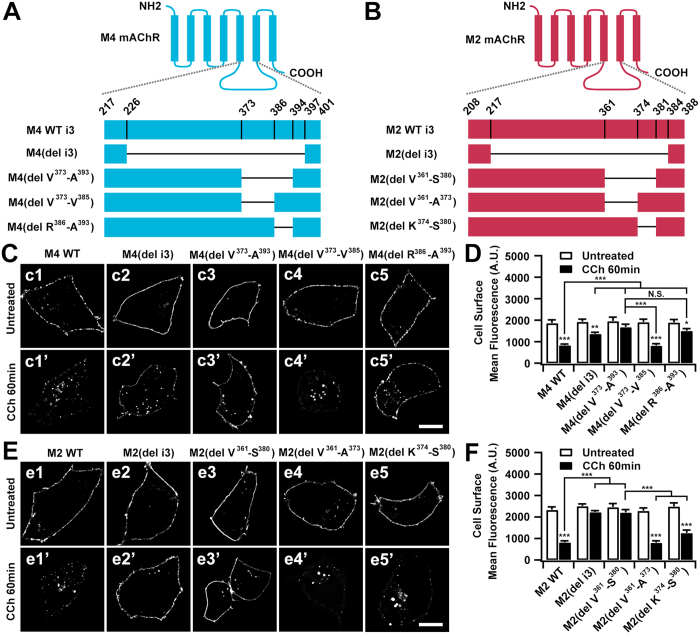
Carboxyl-terminus of i3 is required for internalization of M2 and M4 mAChRs. (**A,B**) Shown are graphic representation of parental and i3 loop-based deletion mutant M4 (**A**) and M2 (**B**) mAChRs. The amino (NH2) and carboxy (COOH) termini of each receptor are positioned on the extracellular and intracellular faces of the plasma membrane, respectively. (**C–F**) Agonist-induced internalization of EGFP-tagged M4 and EGFP-tagged M2 receptors was affected by deleting C-terminus of i3 loop. (**C,E**) Confocal microscopy images of M4-based (**C**) or M2-based (**E**) constructs before or after treatment with CCh (100 μM) for 60 min. (**D,F**) The surface resident receptors in *C* and *E* before or after CCh treatment were quantified. Scale bars, 10 μm. Mean ± SEM of at least 30 cells from at least three independent experiments are shown. *p < 0.05, **p < 0.01, ***p < 0.001. N.S. = non significant.

The i3 loop is known to be critical for agonist-promoted internalization of M2 and M4 mAChRs^15^,^17^,^22^. Here we first examined internalization of the i3 loop-deleted (del i3) M4 or M2 mAChRs in HEK293 cells. As depicted by the schematics in Fig. 3(A,B), truncated M4 (del i3) or M2 (del i3) mutants were constructed with only the membrane-proximal regions of the i3 loops remaining because these regions are thought to be critical for the subtype specific interaction with G-proteins^29^,^30^. Visual inspection and a quantification of plasma membrane-associated fluorescence signals confirmed that there was no substantial decrease of i3-deleted M4 or M2 mAChRs compared with full-length molecules (Fig. 3C, panels c_1_, c_2_; Fig. 3E, panels e_1_, e_2_; Fig. 3D,F), implying that i3-deleted mAChRs were normally expressed and transported to the cell surface. Consistent with previous reports^17^,^22^, after incubation with CCh for 60 min, visible internalization was observed for M4 (del i3) (29%), but at lower levels than that of M4 (55%) (Fig. 3C, panels c_1_′, c_2_′; Fig. 3D). Conversely, internalization of M2 (del i3) was negligible (Fig. 3E, panels e_1_′, e_2_′; Fig. 3F), probably due to loss of the phosphorylation sites for GRK2, which facilitates M2 internalization^31^.

We attempted to define the regions within the i3 loop that are involved in M4 and M2 internalization. Previously a 21 amino acid sequence (V^373^-A^393^) within the carboxyl terminus of i3 was found to be crucial both for internalization and recycling of M4 mAChR in CHO and HEK293 cells^17^,^22^. Here, no obvious internalization of M4 (del V^373^-A^393^) was observed following agonist stimulation (Fig. 3C, panels c_3_, c_3_′; Fig. 3D), confirming the importance of the V^373^-A^393^ sequence in M4 mAChR internalization. To define the specific residues critical for M4 internalization, we divided the 21-residue sequence into amino- and carboxyl-terminal segments and constructed two deletion mutants: M4 (del V^373^-V^385^) and M4 (del R^386^-A^393^). The internalization behavior of M4 (del V^373^-V^385^) was indistinguishable from that of the parental M4 mAChR (Fig. 3C, panels c_1_′, c_4_′; Fig. 3D), while elimination of the carboxyl-terminal segment R^386^-A^393^ substantially impaired the internalization of M4 (del R^386^-A^393^) to a level comparable with M4 (del V^373^-A^393^) (Fig. 3C, panels c_3_′, c_5_′; Fig. 3D). Thus, we apparently identified the essential and nonessential sequences for internalization of the M4 mAChR.

**Figure 4:**
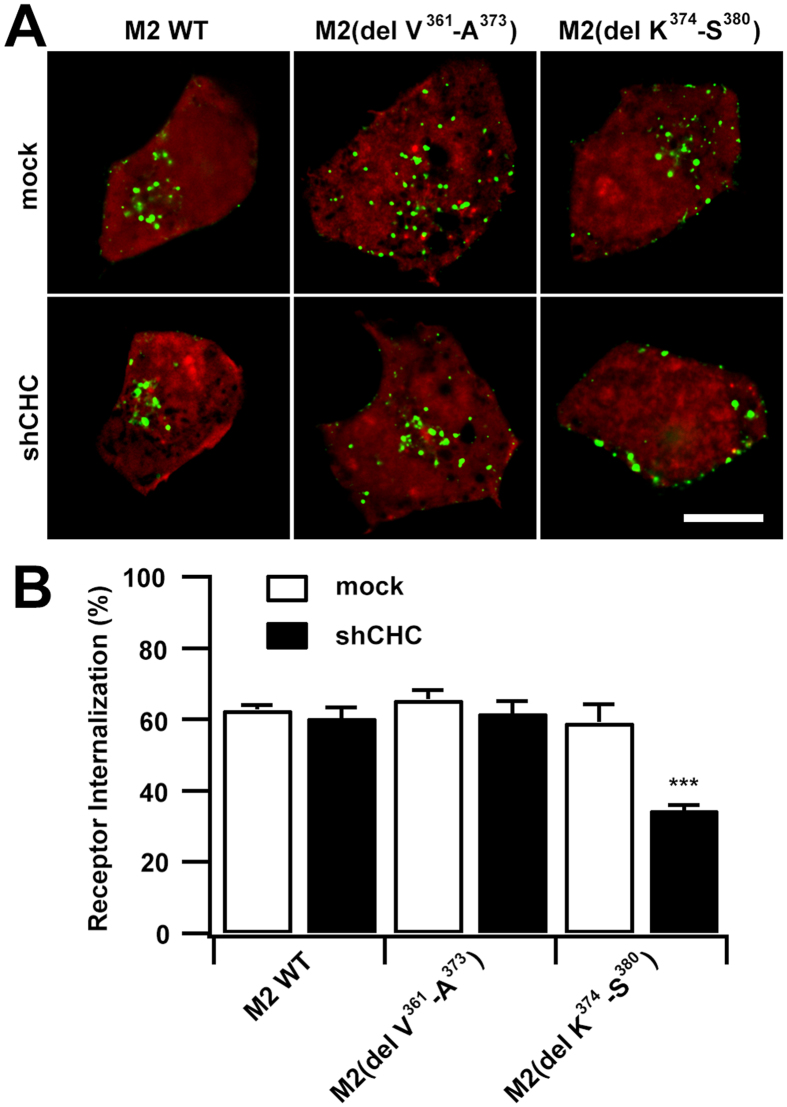
The effects of depletion of clathrin on the internalization of M2 (del V^361^-A^373^) and M2 (del K^374^-S^380^). (**A**) Confocal micrographs show CCh-stimulated internalization of EGFP-tagged M2 WT, M2(del V^361^-A^373^) and M2(del K^374^-S^380^) constructs, respectively. Cells were pre-treated with RFP-tagged vehicle or CHC shRNA for 72 h. (**B**) The extent of internalization was quantified for the constructs in *A* as described in ′′*Methods*′′. Scale bar, 10 μm. Mean ± SEM of at least 30 cells from at least three independent experiments are shown. ***p < 0.001.

To test whether a similar sorting sequence was also located in the i3 loop of M2 mAChR, M2 (del V^361^-S^380^) was developed by deleting the corresponding sequence V^361^-S^380^ (Fig. 3B). Internalization of M2 (del V^361^-S^380^) was not observed after agonist stimulation (Fig. 3E, panels e_3_, e_3_′; Fig. 3F). Two deletion mutants were also constructed, in which the amino-terminus (V^361^-A^373^) and carboxyl-terminus (K^374^-S^380^) of V^361^-S^380^ were deleted (Fig. 3B), respectively. Surprisingly, unlike M4 mAChR, both M2 (del V^361^-A^373^) and M2 (del K^374^-S^380^) were internalized in a normal manner into intracellular compartments after agonist stimulation (Fig. 3E, panels e_4_′, e_5_′; Fig. 3F). To explore the underlying mechanisms of this process, we examined the effects of depletion of clathrin on the internalization of M2 (del V^361^-A^373^) and M2 (del K^374^-S^380^), respectively (Fig. 4). Following CHC knockdown and CCh induction, M2 (del V^361^-A^373^) was internalized normally, consistent with the clathrin-independent mechanism of M2 internalization. In contrast, internalization of M2 (del K^374^-S^380^) was evidently impaired, strongly implying that a clathrin-sensitive mechanism was initiated. Therefore, we propose a model, in which the carboxyl-terminal domain of K^374^-S^380^ functions as a dominant sorting signal and progresses the internalization of M2 mAChR in an unusual clathrin-independent manner, while the upstream adjacent domain of V^361^-A^373^ serves as another sorting signal that targets the receptor toward clathrin-mediated endocytosis, which is normally masked by the downstream dominant signal.

Collectively, the carboxyl-terminal i3 loop of M2 mAChR also contains the sorting signals that determine its internalization pathway. However, in contrast to the short sequence in M4 receptor which simply determines CDE, the regulation of M2 receptor internalization is more sophisticated. A masked motif is found upstream of the CIE signal contained within the carboxyl-terminus and once unmasked, it can switch the M2 mAChR to a CDE pathway.

### Exchanging the endocytic sequences of M2 and M4 mAChRs switches their mode of internalization

**Figure 5:**
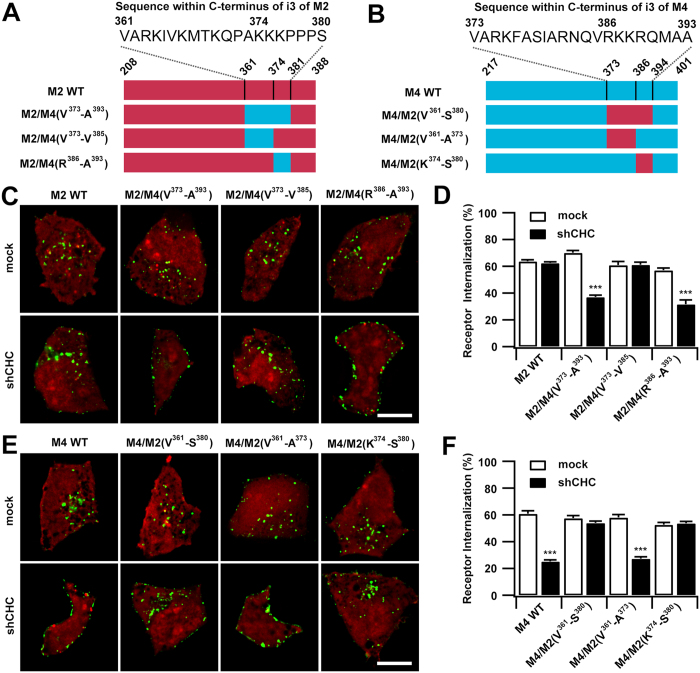
The sequences located in carboxyl-terminal domain of i3 determine the internalization dependence on clathrin for M4 and M2 mAChRs, respectively. (**A,B**) Schematic representations of i3 loop of wild type and chimeric M2 (**A**) and M4 (**B**) mAChRs. The actual sequences of the analogous domains contained within the 20 amino acid for M2 receptor or 21 amino acid region for M4 receptor are shown. (**C,E**) Representative images of EGFP-tagged wild type and chimeric M2 (**C**) or M4 (**E**) mAChRs in response to CCh (60 min; 100 μM). Cells were transfected with RFP-tagged vehicle or CHC shRNA for 72 h. Scale bars, 10 μm. (**D,F**) The levels of internalization for M2-based (**D**) and M4-based (**F**) chimeric mAChRs in cells transfected with vector or shCHC were quantified. Mean ± SEM of at least 30 cells from at least three independent experiments are shown. *** *p* < 0.001.

To verify the sorting sequences identified using deletion mutants, we constructed chimeric M2/M4 mAChRs and analyzed their agonist-induced internalization properties in relation to CDE-dependence. First, we examined the CDE determinant domain of the M4 mAChR using the chimeric receptors M2/M4(V^373^-A^393^), M2/M4(V^373^-V^385^) and M2/M4(R^386^-A^393^), in which the corresponding sequences within the M2 mAChR were truncated and replaced by the M4-derived sequences (Fig. 5A,C,D). Internalization of M2 WT was not affected by the depletion of CHC; however, internalization of M2/M4(V^373^-A^393^) and M2/M4(R^386^-A^393^) were severely impaired, implying that their altered sequences lead the chimeric M2 mAChRs to be processed via a clathrin-sensitive internalization mechanism. In contrast, the internalization of M2/M4(V^373^-V^385^) was normal in the absence of CHC. These results confirmed that the carboxyl-terminal sequence of ^386^RKKRQMAA^393^ within the i3 loop of M4 mAChR is essential to direct it into the clathrin pathway, and is sufficient to confer this property to the clathrin-insensitive M2 mAChR.

We also examined the internalization of the M4-based chimeric mutants M4/M2(V^361^-S^380^), M4/M2(V^361^-A^373^) and M4/M2 (K^374^-S^380^) (Fig. 5B,E,F). The M2-derived sequence V^361^-S^380^ switched the clathrin-dependent internalization of M4 to clathrin-independent mode. A similar tendency was observed for the M4/M2 (K^374^-S^380^) chimera, in which the carboxyl-terminus ^386^RKKRQMAA^393^ of M4 was substituted with the sequence ^374^KKKPPPS^380^ of the M2 mAChR (Fig. 5E,F). These results not only confirm that the ^386^RKKRQMAA^393^ sequence is essential for CDE of M4 mAChRs but also demonstrate the regulation of the carboxyl-terminal ^374^KKKPPPS^380^ for CIE of M2 mAChR. Additionally, CHC depletion completely inhibited the agonist-induced internalization of the M4/M2(V^361^-A^373^) chimera (Fig. 5E,F), implying that this motif is dispensable during sorting of the receptor into the CIE pathway.

### The endocytic sequences are differentially involved in the postendocytic trafficking of M2 and M4 mAChRs

**Figure 6:**
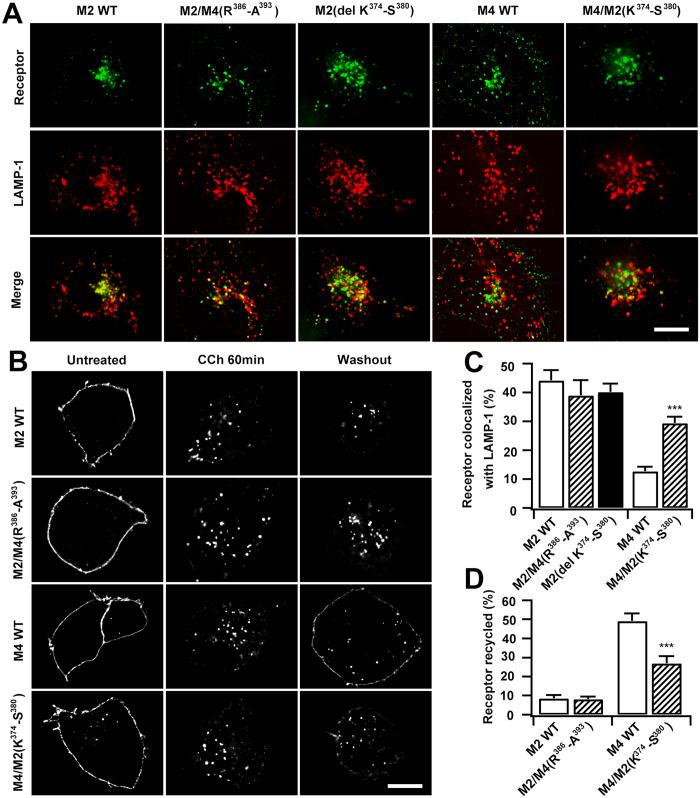
The endocytic sequences are differentially involved in the postendocytic trafficking of M2 and M4 mAChRs. (**A**) Representative immunofluorescences of EGFP-tagged M2, M2/M4(R^386^-A^393^), M2(del K^374^**-**S^380^), M4 or M4/M2(K^374^-S^380^) with respect to the late endocytic and lysosomal marker LAMP-1 in response to CCh (120 min; 100 μM). (**B**) Representative images showing the EGFP-tagged M2, M2/M4(R^386^-A^393^), M4 or M4/M2(K^374^-S^380^) in the absence or presence of CCh (60 min; 100 μM), or followed by 60 min of washout after CCh stimulation. (**C**) The levels of colocalization of various mAChR mutants with LAMP-1 depicted in *A* were determined by Manders coefficient as described in ′′*Methods*′′. (**D**) The recycling of various mAChR mutants depicted in *B* was quantified, as described in ′′*Methods*′′. Scale bars, 10 μm. Mean ± SEM of at least 15 cells from at least three independent experiments are shown. *** *p* < 0.001.

In order to understand how endocytic signals affect the postendocytic trafficking of the mAChR subtypes, we evaluated receptor levels at the lysosomes in cells transfected with EGFP-tagged M2 or M4 mAChRs 120 min after CCh stimulation. Approximately 44% of EGFP-M2 mAChRs were colocalized with the late endocytic and lysosomal marker LAMP-1, while only about 13% of EGFP-M4 mAChRs overlapped with LAMP-1 signals (Fig. 6A,C). Subsequently, a recycling assay was performed for M2- or M4-transfected cells: cells were first stimulated with CCh for 60 min to drive internalization of receptors, and then the plasma membrane-associated receptor level was evaluated 60 min after removing the agonist to allow receptor recycling. The entire process was performed in the presence of leupeptin to inhibit lysosomal degradation and cycloheximide to block newly biosynthesized receptors^21^,^32^. Results showed that M4 mAChRs were significantly recycled back to the plasma membrane (~49%), but M2 mAChRs were not (only 8%; Figs. S2 and 6B, D), consistent with a previous report^26^.

We further examined the postendocytic trafficking of the M2-based chimeric mutant M2/M4(R^386^-A^393^) and the M4-based chimeric mutant M4/M2(K^374^-S^380^). Interestingly, the clathrin-endocytic M2/M4(R^386^-A^393^) was still transported into lysosomes (Fig. 6A,C), whereas an increased fraction of the chimeric M4/M2(K^374^-S^380^) was colocalized with LAMP-1 (29% for M4/M2(K^374^-S^380^) *vs*. 13% for M4 WT; Fig. 6A,C). Consequently, while recycling was not improved for M2/M4(R^386^-A^393^), recycling of M4/M2(K^374^-S^380^) mutant receptors back to the PM markedly decreased (27% *vs*. 49% for M4 WT; Fig. 6B,D), which was due to their trafficking being switched into the lysosomes.

In Figs 3E and 4A, we confirmed that deletion of the sequence K^374^-S^380^ from M2 mAChR unmasked the upstream clathrin sorting signal and resulted in internalization of the mutant receptor via the clathrin pathway. We then examined the localization of the CCh-stimulated M2(del K^374^-S^380^) mutant. The fraction of M2(del K^374^-S^380^) mutant transported into LAMP-1-labeled lysosomes was comparable to that of both M2 WT and the M2/M4(R^386^-A^393^) mutant (Fig. 6A,C). These results suggest that multiple mechanics are located within M2 molecules that ensure their transport into the lysosomes no matter how they enter cells.

### Altering the internalization of mAChRs had no effect on their activity

**Figure 7:**
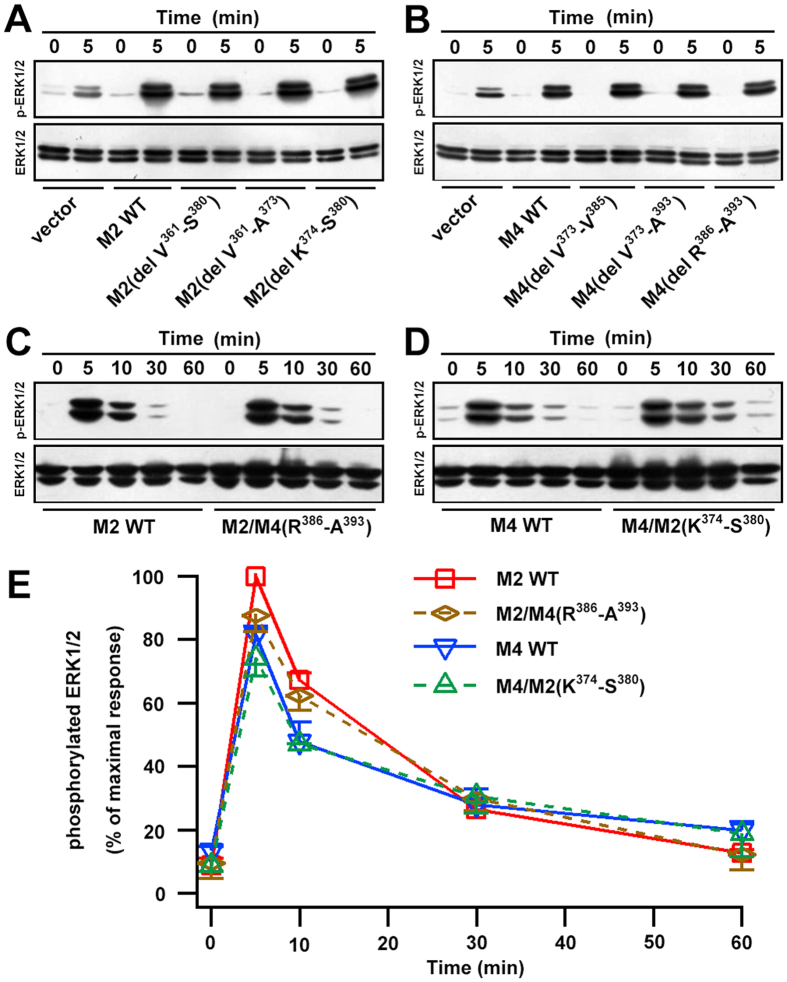
ERK phosphorylation of M2 and M4 mAChRs was not affected by altering the mode of internalization. **(A,B**) Phosphorylation of ERK1/2 was determined by western blotting for HEK293 cells transfected with pcDNA3.1 empty vector, EGFP-tagged wild type and deletion mutants of M2 (A) or M4 (**B**) after treatment with 100 μM CCh for 5 min. (**C,D**) Time courses of phosphorylation of ERK1/2 were determined by western blotting for HEK293 cells transfected with EGFP-tagged M2 and M2-based chimeric M2/M4(R^386^-A^393^) (**C**) or M4 and M4-based chimeric M4/M2(K^374^-S^380^) (**D**). Cells were treated with 100 μM CCh for indicated time points as depicted. Total ERK was used as a loading control. (**E**) The densitometric analysis of ERK 1/2 phosphorylation elicited by the activated receptor depicted in *C* and *D* was normalized to the maximum value and plotted. Mean ± SEM of immunoblots in three independent experiments are shown.

In previous reports, stimulation of mAChRs by cholinergic agonists caused time- and dose-dependent increases in extracellular signal-regulated kinase (ERK) phosphorylation in various cell types, including HEK293 cells^19^,^33^,^34^. To understand whether the mode of internalization mAChRs affected their activity, the downstream signaling of the MAPK pathway following mAChRs activation was assessed via measurement of ERK 1/2 phosphorylation (Fig. 7). CCh-stimulated M2 and M4 mAChRs both caused substantial ERK phosphorylation, and the activation process was time-dependent. With prolonged CCh stimulation, ERK1/2 phosphorylation levels decreased compared with the peak values at 5 min. In addition, M2-expressing cells exhibited higher level of ERK1/2 phosphorylation than the M4-expressing group (Fig. 7E), probably due to the higher expression levels observed for M2 mAChRs (Fig. 1C).

The signal activity of M2- and M4-based deletion mutants was found to be normal by examining their ERK phosphorylation levels 5 min after stimulation (Fig. 7A,B). Cells were then transfected with chimeric M2/M4(R^386^-A^393^), which took the CDE pathway, or M4/M2(K^374^**-**S^380^), which took the CIE pathway. Following CCh stimulation, the ERK phosphorylation levels of each chimera were comparable to the corresponding wild-type mAChR subtype (Fig. 7C–E). Taken together, these results suggest that agonist-induced phosphorylation of ERK elicited by M2 or M4 mAChR activation in HEK293 cells is not compromised by the distinct endocytic properties of mAChRs.

## Discussion

An accumulation of evidence has revealed the molecular details and regulation of clathrin-endocytic GPCRs, while the molecular mechanisms of nonclathrin-endocytic GPCRs are less clear^3^,^35^. In this study, we investigated agonist-stimulated internalization and intracellular transport of two structurally and functionally homologous mAChRs, M2 and M4. Our results support the hypothesis that the preferred internalization pathway for activated M2 mAChR is clathrin-independent. We identified a specific sorting signal that resided in the carboxyl-terminal port of the i3 loop, which distinguishes M2 from M4 and other mAChR subtypes by directing it to the CIE pathway and finally to lysosomes. We also demonstrated that CDE alternatively serves to internalize M2 mAChRs if the predominant CIE process is interrupted.

Previous studies indicate that the cytoplasmic C-tails of many GPCRs contain the sorting sequences that control their endocytosis or post-endocytic trafficking^11^,^36^. Muscarinic receptors are distinct from most GPCRs because they possess a short carboxyl-terminus that lacks specific endocytic sorting signals but has a particularly long i3 loop^13^; sorting signals responsible for recognition by G proteins^30^,^37^ or endocytic machinery^38^,^39^ and sites of phosphorylation^31^,^40^ have been identified within this region. Here, we showed that the i3 loop within the structurally similar M2 and M4 mAChRs contained the key residues that determined their fate. Indeed, M4 mAChR residues 373–393 correspond to the amino acid sequence VARKFASIARNQVRKKRQMAA, which was previously suggested as important for receptor recycling^22^. We demonstrated that only the eight carboxyl-terminal residues ^386^RKKRQMAA^393^ within this region serve as the indispensable sorting sequence for M4 mAChR endocytosis. When the corresponding region in M2 mAChR was replaced with this sequence, the endocytosis of the chimeric M2/M4 (R^386^-A^393^) mAChR was also switched to a clathrin-sensitive mode. Furthermore, we showed that residues ^361^VARKIVKMTKQPAKKKPPPS^380^ of the M2 mAChR corresponded to the 21-amino acid sequence 373-393 within the M4 mAChR and did not belong to any previously defined endocytic signal in mAChRs. We found that these 20 amino acid residues are specifically important for the endocytosis of M2 mAChRs. The downstream domain^374^KKKPPPS^380^ serves as a dominant CIE signal for M2 mAChRs; surprisingly, the upstream domain ^361^VARKIVKMTKQPA^373^ acts as a signal for CDE. Given normal conditions, the M2 mAChRs are sorted into the CIE pathway; however, following removal of the downstream dominant signal, V^361^-A^373^ is unmasked and directs the receptor to a clathrin-mediated internalization pathway. This alternative mechanism for internalization of M2 mAChRs is probably an adaptive strategy of the cells in response to sophisticated environments. Schlador *et al*. previously identified three regions within the M2 mAChR that cooperate to specify the receptor’s dynamin-independent endocytic profile^41^. Taken together, these results suggest that internalization of M2 mAChRs may involve a more complex process; therefore, further investigation will be necessary to identify the structural requirements for entry into the clathrin-independent pathway and elucidate the underlying mechanisms of the process.

Interestingly, the analogous sequences of M2-derived ^374^KKKPPPS^380^ and M4-derived ^386^RKKRQMAA^393^ are differentially involved in the post-endocytic trafficking of the two mAChR subtypes, although they both play important roles in determining the internalization properties of receptors. On one hand, the endocytic sequence of M4-derived ^386^RKKRQMAA^393^ converted the internalization of M2 receptors from the clathrin-independent to -dependent mode, but it cannot change the receptors’ destination of targeting to lysosomes, that is, the M2 receptor was neither switched into recycling pathway nor accumulated in the sorting endosome. Considering the unaltered lysosomal localization of M2(del K^374^-S^380^) and Hashimoto’s results that only when grafting the M4 mAChR-derived sequence V^373^-A^393^ into a whole i3-truncated M2 mAChR was a recycling chimeric M2 mAChR produced^22^, we believe that other mechanics located in the i3 loop of M2 mAChR, which ensures the lysosomal transport of the activated M2, no matter it is internalized through normal CIE pathway or through CDE led either by the unmasked CDE endocytic signal, like M2(del K^374^-S^380^), or by a grafted CDE endocytic signal, like M2/M4(R^386^-A^393^). On the other hand, deletion of the motif ^386^RKKRQMAA^393^ from M4 caused the M4(del R^386^-A^393^) unable to internalize, however, switching the M2-derived motif ^374^KKKPPPS^380^ into M4 not only caused the M4/M2(K^374^-S^380^) chimera to internalize via clathrin-independent pathway, but also resulted in increased routing of the chimera toward the lysosomal pathway. Presently it is unclear about whether and how the motif ^374^KKKPPPS^380^ affects the lysosomal targeting, future work is needed to clarify whether it functions autonomously or cooperatively with other mechanics within the i3 loop, and to clarify whether it also works effectively in other clathrin-endocytic M1, M3 and M5 mAChR subtypes and even other remote-related GPCRs.

Many GPCRs are internalized through clathrin-mediated endocytosis; and the components of the underlying machinery have largely been identified. Sorting signals in the intracytosolic domains of GPCRs that regulate trafficking through the endosomal-lysosomal system include the following: tyrosine- and dileucine-based motifs and PDZ ligands that are recognized by distinct endocytic adaptor proteins; covalent modification of GPCRs, such as β2AR and CXCR4, with ubiquitin, which serves as a signal for internalization and/or lysosomal sorting^3^,^36^,^42^. Unfortunately, due to the difficulties involved in investigating CIE pathways, our knowledge of endocytic mechanics, post-endocytic trafficking and their regulation is not well developed as for CDE. In this study, we did not identify the endocytic machinery protein recognized by ^374^KKKPPPS^380^, further investigation, probably involving yeast two-hybrid or co-immunoprecipitation screening combined with mass spectroscopy identification, will be required to achieve this. Additionally, as we know, lysine residues function as ubiquitin-acceptors^36^, further work will be tempting to identify whether this sequence is involved in ubiquitination and if so, whether this has a role in internalization or lysosomal sorting. We believe these future works will surely improve our understanding of sequence-dependent clathrin-independent endocytosis and trafficking.

In this study, the ERK phosphorylation response elicited by mAChRs was used to examine the effects of different internalization modes on downstream signaling. We confirmed that the temporal activation of ERK1/2 by the wild-type and chimeric M2 or M4 mAChRs was similar. These results suggest that the altered internalization modes of the mAChR subtypes had no direct effect on their distinct downstream signaling, possibly because the rate of receptor internalization was unchanged and therefore the coupling of the mAChRs with G proteins was unaffected^43^,^44^. Notably, the M2 mAChR acts as an autoreceptor at presynapes in modulating neurotransmitter release^45^,^46^. The selective CIE pathway of M2 mAChRs is speculated to efficiently separate them from synaptic vesicle-associated components, which are presumably internalized primarily via the CDE pathway after exocytosis^47^,^48^. In future research, it will be valuable to identify whether altering the internalization mode compromises other signaling events elicited by activated mAChRs. Identifying the other cellular physiological functions that are coupled to these distinct receptor trafficking modes will also be important. Additionally, we believe increasing our understanding of the mechanisms that underlie the endocytosis and postendocytic trafficking of mAChRs and selective modulation of sorting pathways could have important implications for drug development.

In summary, the i3 loop is important for the functions of M2 and other mAChRs. Previous studies demonstrated that the amino-terminal residues within the i3 loop determine the M2 mAChR specificity for G proteins^37^ and that the central region contains the sites for phosphorylation and arrestin-binding^19^. Here, we demonstrated a novel sorting sequence located in the carboxyl-terminal portion of the i3 loop that determines CIE of the M2 mAChR; thus, we revealed a previously unidentified mechanism for mAChR trafficking.

## Methods

### Materials

Alexa 568-conjugated transferrin (Tfn) was purchased from Invitrogen (Carlsbad, CA, USA). Carbachol (CCh) was from Sigma (St. Louis, MO, USA) and cycloheximide was from Aladdin-reagent Inc. (Shanghai, China). Mouse anti-LAMP-1 antibody (H4A3) was purchased from the Developmental Studies Hybridoma Bank (DSHB, University of Iowa, IA, USA) and Cy3-conjugated donkey-anti-mouse secondary antibody was from Jackson ImmunoResearch (West Grove, PA, USA). Rabbit polyclonal pERK, ERK antibodies, and anti-rabbit IgG horseradish peroxidase-conjugated secondary antibody were from Cell Signaling Technology (Danvers, MA, USA). Anti-HA monoclonal antibody conjugated with horseradish peroxidase (clone3F10) was from Roche Bioscience (Basel, Switzerland). Chemiluminescence reagents were from Pierce Protein Research Products (Rockford, IL, USA).

**Plasmid constructs.** The plasmid ss-EGFP-M2 (ss, signal sequence) was a kind gift from James W. Wells (University of Toronto), and ss-TagRFP-M2 was created by replacing EGFP with TagRFP. ss-EGFP-M4 was generated by replacing M2 with M4, which was amplified from cDNA of human brain.

Six EGFP-M2- or EGFP-M4-derived deletion mutants: M2 (del V^361^-S^380^), M2 (del V^361^-A^373^), M2 (del K^374^-S^380^), M4 (del V^373^-A^393^), M4 (del V^373^-V^385^) and M4 (del R^386^-A^393^), were generated by PCR overlap extension method^49^ using ss-EGFP-M2 or ss-EGFP-M4 as templates, respectively. The chimeric mAChR receptors between M2 and M4, M2/M4(V^373^-A^393^), M2/M4(V^373^-V^385^), M2/M4(R^386^-A^393^), M4/M2(V^361^-S^380^), M4/M2(V^361^-A^373^) and M4/M2(K^374^-S^380^), in which the regions of C-termini of i3 were exchanged, were produced by PCR overlap extension method. To examine mAChR expression by ELISA, the HA-tagged chimera were generated by inserting HA epitope (YPYDVPDYA) between the signal sequence and EGFP by PCR-based insertion mutagenesis using ss-EGFP-M2 or ssEGFP-M4 as a template.

The 21–nucleotide target sequence of the human *chc* gene (5′-taatccaattcgaagaccaat-3′)^28^ was inserted into the pRNAT-H1.1-shutter/GFP or pRNAT-H1.1-shutter/RFP vector, which was modified with the template of pRNAT-H1.1-shutter/GFP. All constructs were verified by DNA sequencing.

### Cell culture and transfections

Human embryonic kidney-derived (HEK) 293 cells were grown in DMEM (Gibco) supplemented with 10% fetal calf serum (Gibco) and 1% penicillin–streptomycin at 37 °C in a humidified atmosphere of 5% CO_2_. Transfections were performed using Lipofectamine 2000 (Invitrogen) in 12-well-plates according to the manufacturer’s protocol, and cells were examined 24–48 h after. shRNA-transfected cells were used 72 h after transfection.

### Receptor internalization assay

To induce mAChR internalization, transfected HEK293 cells were incubated with the muscarinic receptor agonist carbachol (100 μM) in serum-free medium (α-MEM without phenol red supplemented with 20 mM Hepes pH 7.4 and 0.1% BSA) at 37 °C for various periods of time as indicated. Then, cells were washed extensively on ice with ice-cold serum-free medium thrice, fixed with 3.7% PFA for 15 min at room temperature and mounted with the mounting solution. The internalization was examined by confocal imaging or ELISA. For Tfn uptake experiments, cells were incubated in serum-free medium containing 10 μg/ml Alexa 568-Tfn at 37 °C for 15 min.

### Receptor recycling assay

HEK 293 cells were transfected with each subtype of muscarinic receptor WT or mutant. Before CCh incubation, cells were pretreated with medium containing cycloheximide (20 μg/ml) and leupeptin (100 μM) for 30 min to eliminate newly synthesized mAChRs^26^ and inhibit lysosomal proteolysis of internalized mAChRs^50^. Cells were incubated with 100 μM CCh for 60 min to induce mAChR internalization. Then, cells were washed twice with DMEM and incubated in fresh medium for 60 min. Cycloheximide and leupeptin were included in whole procedure.

### Immunofluorescence staining and confocal imaging

Visualization of LAMP-1 was performed using immunofluorescence staining. Briefly, Cells grown on coverglass were fixed for 10 min with 3.7% paraformaldehyde and treated with block and permeabilizing buffer (PBS containing 2% BSA and 0.2% saponin) for 30 min at RT. Subsequently, cells were washed several times with PBS and incubated with mouse anti-LAMP-1 antibody (1:1000) for 30 min at RT. After wash heavily with PBS, cells were incubated with Cy3-conjugated donkey-anti-mouse secondary antiboy (1:600) for 30 min at RT. Secondary antibody was washed out and coverglass was mounted onto slide for fluorescence imaging. A Spinning-disk confocal imaging system (CSU-X1 Nipkow Yokogawa, Japan) with Andor IQ 1.8 software (Andor Technology plc, Springvale Business Park, United Kingdom) was used for fluorescent imaging. An Olympus IX-71 inverted microscope (Olympus Corp., Japan) was equipped with an oil-immersion objective (150 × NA 1.45 or 100 × NA 1.3). A CO_2_ and 37 °C temperature-controlled incubator (Tokai Hit, Japan) was used for live cell imaging. 14-bit digital images were acquired with an EM CCD camera (DU897, ANDOR iXon, United Kingdom). An Andor LC-401A Laser Combiner with diode-pumped solid state (DPSS) lasers was used to excite GFP/EGFP and RFP/TdimerII/mCherry/Alexa 568 at 491 and 561 nm, respectively.

### Confocal data analysis

Images were deconvolved using AutoQuant X software (Media Cybernetics Inc., MD) and further processed by means of ImageJ (NIH). Linear profile analysis for single cell was done using ImageJ plugin “Plot Profile”. The extent of internalization at different time intervals upon agonist induction or recycling at 60 min of washout following 60 min of CCh-induction was calculated. To do so, a 10-pixel-wide line segment was drawn along the contour of each cell to calculate the intensity of plasma membrane-associated signals, which was subtracted by the background signals. The extent of the mAChRs internalization was quantified by calculating the average intensity of the plasma membrane-associated tagged mAChRs at different time intervals after CCh-induction and normalized with respect to the signals of control conditions, that is, the intensity of membrane associated mAChRs before agonist stimulation (I_untreated_). The net recycling of mAChRs was defined as 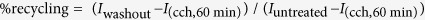, in which I_washout_ means the PM-associated mAChR signal intensity after 60 min of CCh-induced internalization and subsequent 60 min of chasing for recycling, I_(CCh, 60 min)_ means the PM-associated mAChR signal intensity after 60 min of CCh-induction^51^.

For quantification of colocalization, images were deconvolved by AutoQuant. Background was subtracted using the rolling-ball subtraction algorithm with the radius set to 50 pixels. Colocalization analysis was done using ImageJ plugin “JACop”. Colocalization between EGFP-M4 and TagRFP-M2 was determined by Pearson’s coefficient. The percentage of overlapped area between EGFP-mAChR signals over mCherry-Rab5 or LAMP-1 signals was determined by Manders coefficient.

### Quantification of receptor expression and internalization by ELISA

For quantification of cell surface and total receptor expression, ELISA assays were performed as described^52^. 8 × 10^5^ HEK293 cells grown overnight with 80–90% confluence in 12-well plates were transfected with 0.3 μg of pcDNA3.1 (as control) or HA-tagged M2 or M4 mAChR plasmids and replated into 96-well plates. 48 h after transfection, cells were fixed with 4% paraformaldehyde in PBS (non-permeabilized buffer, for measuring the cell surface level of mAChRs) or fixed with 4% paraformaldehyde and 0.1% Triton-100 in PBS (permeabilized buffer, for measuring the total expression of mAChRs). After that, cells were incubated with anti-HA monoclonal antibody for 30 min at room temperature and then extensively washed. Bound antibody was measured by chemoluminescence using SuperSignal substrate (Pierce Protein Research Products) and a 2103 EnVision™ Multilabel Plate Readers (Perkin Elmer, Waltham, MA). For internalization assay, 48 h after transfection, cells were incubated with 100 μM CCh for the indicated periods of time at 37 °C. After fixation with 4% paraformaldehyde in PBS, the cell surface level of HA-tagged mAChRs were examined as described above, and the values were normalized to that of untreated cells. Estimates of the rate constants for receptor internalization were performed by fitting data using a single-phase exponential decay equation (Igor Pro software).

### ERK1/2 phosphorylation assay

HEK293 cells expressing HA-tagged various versions of mAChR plasmid or control vector were plated into 6-well plates. Cells were simulated with 100 μM CCh for the indicated time periods. After lysis on ice using ice-cold lysis buffer, cell lysates were sonicated, and protein content was measured by the Bradford method (Bio-Rad Laboratories Ltd., Hertfordshire, UK). 20 μg of cellular extracts were boiled in SDS sample loading buffer (0.0625 M Tris pH 6.8, 2% SDS, 10% glycerol, 0.1 M DTT, 0.01% bromophenol blue) for 5 min and subjected to 12% SDS-PAGE followed by transferring to nitrocellulose membranes (Millipore, Bedford, MA) for immunoblotting. Prior to antibody incubation, membranes were blocked in blocking buffer (5% nonfat dry milk in TBS and 0.1% Tween 20) for 1 h at room temperature. Phospho-p42/44 ERK1/2 and p42/44 ERK1/2 antibodies were incubated overnight at 4 °C, 1/2000 diluted. Membranes were then washed thrice with TBS-tween, incubated with an anti-rabbit IgG horseradish peroxidase-conjugated secondary antibody (1:20000) for 2 h at room temperature, and were washed extensively with TBS-tween before chemiluminescent detection was performed using X-ray films. Band intensity was measured using ImageJ.

### Data analysis

Averaged results were presented as the mean value ± SEM. Significance values (*p* values) were calculated using either Student’s *t*-test or Mann-Whitney rank sum test as appropriate. Data with a *p* value < 0.05 was considered significant. Asterisks *, ** and *** denote statistical significance with *p* values less than 0.05, 0.01 and 0.001, respectively.

## Additional Information

**How to cite this article**: Wan, M. *et al*. Unraveling a molecular determinant for clathrin-independent internalization of the M2 muscarinic acetylcholine receptor. *Sci. Rep*. **5**, 11408; doi: 10.1038/srep11408 (2015).

## Supplementary Material

Supplementary Information
