# Supplementary material for: Unraveling a molecular determinant for clathrin-independent internalization of the M2 muscarinic acetylcholine receptor
Source: Sci Rep. 2015 Jun 22;5:11408. doi: 10.1038/srep11408 (PMC4476042; doi:10.1038/srep11408)
Supplement: Supplementary Information [file srep11408-s1.pdf]

# Supplementary Information

## Unraveling a molecular determinant for clathrin-independent internalization of the M2 muscarinic acetylcholine receptor

Min Wan[1,*], Wenhua Zhang[1,*], Yangli Tian[1], Chanjuan Xu[1], Tao Xu[1,2], Jianfeng Liu[1,†], and Rongying Zhang[1,†]

[1]Key Laboratory of Molecular Biophysics of the Ministry of Education, College of Life Science and Technology, Huazhong University of Science and Technology, Wuhan, Hubei, China

[2]National Laboratory of Biomacromolecules, Institute of Biophysics, Chinese Academy of Sciences, Beijing, China

*These authors contributed equally to this work.

[†]To whom correspondence should be addressed. E-mail: ryzhang@hust.edu.cn or jfliu@mail.hust.edu.cn

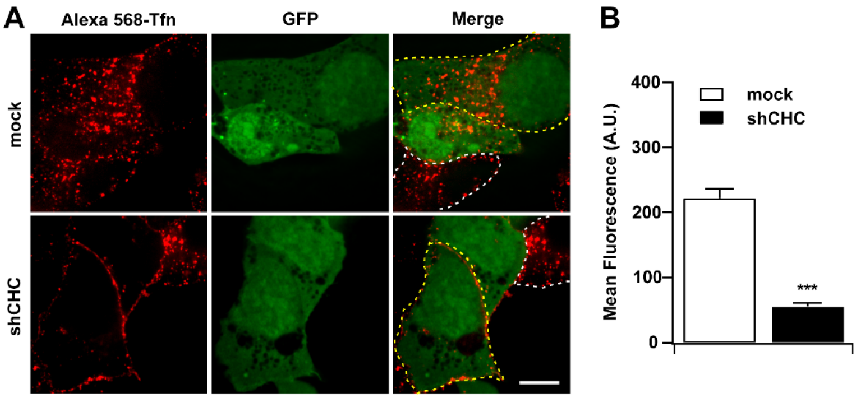


**Figure S1 | CHC-depletion blocked uptake of Tfn in HEK293 cells.** (A) Representative images of Alexa 568-Tfn distribution in cells transfected with GFP-tagged vector or shCHC. Alexa 568-Tfn uptake experiments were performed as described in *"Methods"*. Cell contours were outlined, with yellow dash lines showing transfection-positive cells and white dash lines showing transfection-negative cells. Scale bar, 10 μm. (B) Quantification of Tfn uptake in cells transfected with vector or shCHC. Mean ± SEM of at least 40 cells from at least three independent experiments are shown. ***$p < 0.001$.

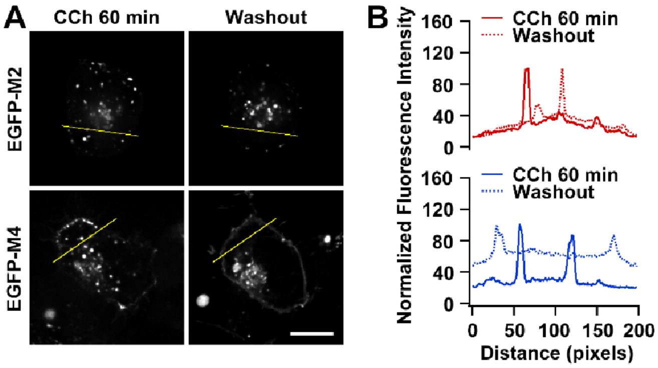


**Figure S2 | Analysis of EGFP-tagged M2 and M4 mAChR recycling in living HEK293 cells.** (A) Representative live imaging of cells stimulated with CCh (100 μM) and subsequent washout of 60 min. Scale bar, 10 μm. (B) Linear profile analysis showed after washout for 60 min, M2 signals were still located intracellularly due to CCh-induction, but the M4 signals partially trans-located onto the plasma membrane.
